# Peripapillary Choroidal Thickness and Open-Angle Glaucoma: A Meta-Analysis

**DOI:** 10.1155/2016/5484568

**Published:** 2016-05-19

**Authors:** Zhongjing Lin, Shouyue Huang, Bing Xie, Yisheng Zhong

**Affiliations:** Department of Ophthalmology, Ruijin Hospital Affiliated Medical School, Shanghai Jiaotong University, Shanghai 200025, China

## Abstract

*Purpose*. To investigate the potential relationship between open-angle glaucoma (OAG) and peripapillary choroidal thickness (PPCT).* Materials and Methods*. Relevant publications were searched systematically through various databases from inception to January 2016. Studies comparing PPCT in OAG patients and healthy controls were retrieved. All qualified articles were analyzed using Stata 14.0 and Revman 5.3 software.* Results*. A total of 13 studies were identified for inclusion. There was a significant reduction of average PPCT in OAG patients compared to control participants (WMD = −24.07, 95% CI: −34.29, −13.85). Reduction of PPCT was significant in the superior (WMD = −28.87, 95% CI: −44.96, −12.78) and nasal (WMD = −21.75, 95% CI: −41.52, −1.98) sectors, but there was no significant reduction of PPCT in the inferior (WMD = −9.57, 95% CI: −36.55, 17.40) and temporal (WMD = −13.85, 95% CI: −35.40, 7.70) sectors. No obvious publication bias was detected.* Conclusions*. This meta-analysis suggests that open-angle glaucoma patients have significantly decreased peripapillary choroidal thickness compared to healthy individuals. Peripapillary choroidal thickness measured by optical coherence tomography may be an important parameter to consider in open-angle glaucoma.

## 1. Introduction 

Glaucoma is becoming more common than expected, which is characterized by loss of retinal nerve fiber layers and an associated change in visual field, resulting in irreversible blindness worldwide. The total number of people aged 40–80 years diagnosed as having primary open-angle glaucoma (POAG) is predicted to increase to 79.76 million in 2040, approximately 85% of the glaucomatous population [1]. The pathogenesis of open-angle glaucoma (OAG) has not been fully interpreted yet and accumulating evidence suggests that it is associated with the reduced blood perfusion to the optic nerve [2–4]. As the peripapillary choroid branches are the main source of blood supply to this region, it has been proposed that an abnormal choroid circulation could be involved in the occurrence of glaucomatous optic neuropathy. However, it is specifically challenging to study because it is located beneath the retinal pigment epithelium (RPE).

A precise clinical assessment of choroidal changes might be particularly important for an accurate interpretation of glaucoma. Prior to the improvements of optical coherence tomography (OCT), the choroid could only be evaluated by indocyanine green angiography (ICGA) [5], laser Doppler flowmetry [6], and ultrasound [7], all of which are not sufficient to examine the choroid in detail. Optical coherence tomography offers the opportunity of providing a relatively detailed quantitative measurement tool for choroidal structure at a range of locations across the posterior pole with high-quality and cross-sectional images [8, 9]. An estimate of choroidal thickness can be obtained by determining the distance from RPE/Bruch's membrane interface to sclerochoroidal interface.

With renewed interest in the potential role of the choroid in the pathophysiology of OAG, some recent studies have explored PPCT measured by OCT in OAG patients, only to find conflicting results. If PPCT changes correlate with OAG, evaluation of PPCT would be particularly important, because earlier detection and better monitoring of glaucoma would minimize the risk of blindness. To determine whether PPCT changes in OAG patients or not, we therefore reviewed the current literature and performed a meta-analysis.

## 2. Materials and Methods

This updated meta-analysis was conducted under the guidance of the Preferred Reporting Items for Systematic Reviews and Meta-Analyses (PRISMA) statement (see Checklist S1 in Supplementary Material available online at http://dx.doi.org/10.1155/2016/5484568) [10].

### 2.1. Literature Search

An initial systematic search of PubMed, EMBASE, ISI Web of Knowledge, and the Cochrane Library was conducted without language or time restrictions. The final search was performed in January 2016. Systematic searches were conducted using the following key words in different combinations: “peripapillary choroidal thickness,” “optical coherence tomography,” and “open-angle glaucoma.” In addition, the reviewers also went through the reference lists of relevant published articles manually for any additional study.

### 2.2. Inclusion and Exclusion Criteria

Published studies were included if they were in cross-sectional or case-control design comparing the differences in peripapillary choroidal thickness measured by OCT between patients with OAG and healthy controls. Abstracts from conferences, case reports, duplicate publications, letters, and reviews were excluded.

### 2.3. Data Extraction

Two review authors extracted all the required data independently from the included articles. Divergences were eliminated by discussion. The extracted contents included the following: first author, publication year, location, OCT type, study size, mean age, mean axial length, IOP at imaging, and mean visual field MD. The peripapillary choroidal thickness parameters evaluated were average, superior, inferior, nasal, and temporal thickness. Superior choroidal thickness was defined as choroidal thickness measured at a certain location superior to the center of optic nerve head or the mean value of several different points in this sector. Similarly, we used this method to extract the inferior, nasal, and temporal choroidal thickness.

### 2.4. Quality Assessment

The Newcastle-Ottawa Scale (NOS) was employed in the quality assessment in our meta-analysis [11]. This quality scoring system ranging between zero up to nine stars contains three broad perspectives, divided into 8 items specifically. A score of 6 or higher indicates that the study has adequate quality. Two review authors subjectively scored each included study and any differences were resolved by discussion.

### 2.5. Statistical Analysis

Statistical analysis was performed using Revman software (version 5.3; Cochrane Collaboration, Oxford, United Kingdom). As the PPCT was continuous outcomes, the effect sizes were measured using the weighted mean difference (WMD) and 95% confidence interval (CI). We examined heterogeneity among the studies using the Chi-square test and *I* ^2^ test. *P* < 0.05 for Chi-square test or *I* ^2^ > 50% represented the presence of obvious heterogeneity; then a random-effect analysis model was used and subgroup analysis would be conducted. Otherwise, the fix-effect analysis model was applied. *P* < 0.05 represented a statistically significant difference for overall effect.

### 2.6. Sensitivity Analysis

To explore the stability and reliability of our results, we performed sensitivity analysis using Stata (version 14; StataCorp, College Station, Texas). This was conducted by deleting one study successively and recalculating the effect sizes of the remaining studies.

### 2.7. Publication Bias

In order to detect potential publication bias, funnel plots were performed using Revman 5.3. Meanwhile, Begg's and Egger's tests were also calculated for the primary outcome using Stata (version 14; StataCorp, College Station, Texas).

## 3. Results

### 3.1. Literature Search

**Figure 1:**
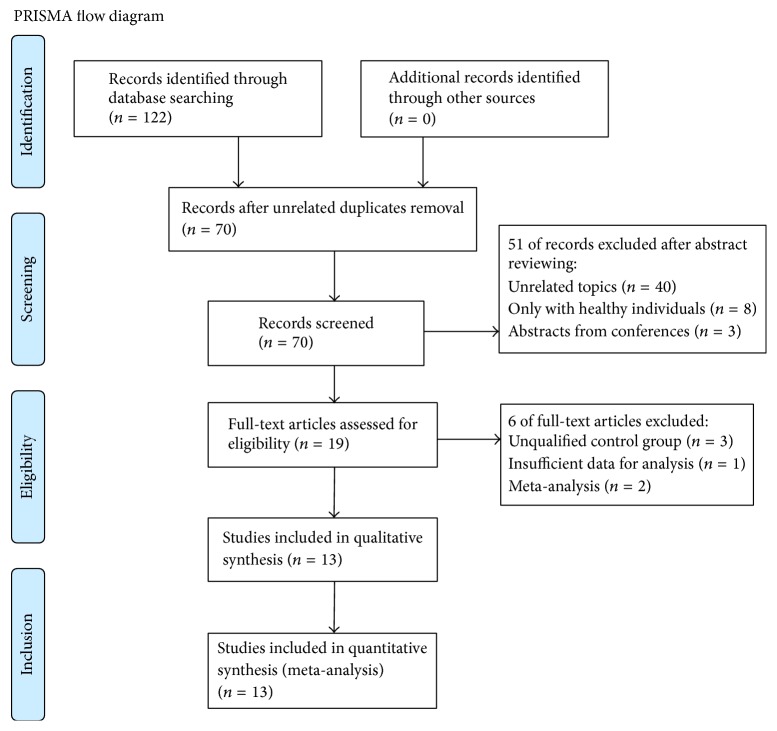
Flow diagram of the selection process in the meta-analysis.

We initially identified 122 articles from the databases and no additional studies were identified. The majority of these were excluded after the application of inclusion and exclusion criteria, mainly because most of them were not relevant to our analysis. The remaining 19 articles were subsequently reviewed in detail. Six studies were finally excluded for various reasons: three due to unqualified control groups (two using glaucoma suspects [12, 13] and one using the collateral nonglaucomatous eyes [14]), one due to insufficient data which just provided the mean value without the SD [15], and the other two were meta-analyses [16, 17]. The remaining 13 studies were eventually selected for our meta-analysis.  Figure 1 showed the flow diagram of the search results.

### 3.2. Study Characteristics and Quality Assessment

The detailed characteristics of the included studies were summarized in Table 1. Four were conducted in Korea [18–21], 2 were conducted each in America [22, 23], Japan [24, 25], and China [26, 27], and 1 was conducted in Canada [28], Germany [29], and Belgium [30]. Various OCT instruments were applied in these studies, such as Heidelberg (Heidelberg Engineering, Heidelberg, Germany), RTVue-100 SD-OCT (Optovue Inc., Fremont, CA), Cirrus HD-OCT (Carl Zeiss Meditec, Dublin, CA), and swept-source OCT (SS-OCT). With regard to the quality assessment, Table 2 shows the quality score of each included article using the Newcastle-Ottawa Scale. All the studies had a score of 6 or higher, suggesting a low risk of bias.

### 3.3. Efficacy Analysis

#### 3.3.1. Open-Angle Glaucoma and Average PPCT

**Figure 2:**
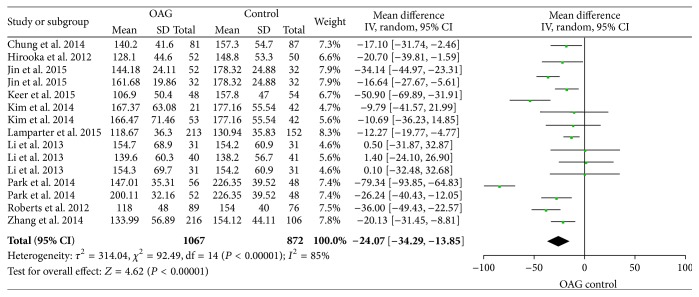
Forest plots of average PPCT between open-angle glaucoma patients and controls.

There was significant heterogeneity in the analysis of average PPCT between OAG and the control group (*χ* ^2^ = 92.49, *P* < 0.05, *I* ^2^ = 85%) and random-effects model was applied. The result showed that the average PPCT in OAG patients was reduced significantly compared to the healthy individuals (WMD = −24.07, 95% CI: −34.29, −13.85) (Figure 2).

#### 3.3.2. Open-Angle Glaucoma and 4-Quadrant PPCT

**Figure 3:**
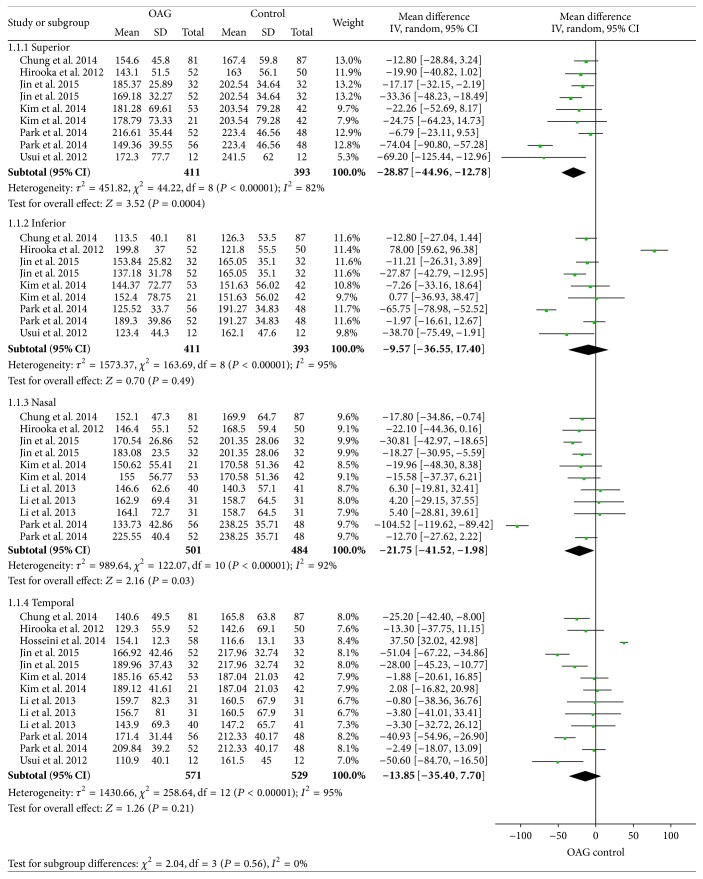
Forest plots of PPCT in each quadrant between open-angle glaucoma patients and controls.

Moreover, PPCT in each sector between the two groups was used for meta-analysis. The results revealed that there was particularly apparent heterogeneity among these studies: superior (*I* ^2^ = 82%), inferior (*I* ^2^ = 95%), nasal (*I* ^2^ = 92%), and temporal (*I* ^2^ = 95%). However, meta-analysis of each sector showed that a significant reduction of PPCT between the two groups in the superior (WMD = −28.87, 95% CI: −44.96, −12.78) and nasal (WMD = −21.75, 95% CI: −41.52, −1.98) parts was identified, but PPCT in the inferior (WMD = −9.57, 95% CI: −36.55, 17.40) and temporal (WMD = −13.85, 95% CI: −35.40, 7.70) sectors was not significantly different in OAG patients compared to the control group (Figure 3).

#### 3.3.3. Subgroup Analysis

**Figure 4:**
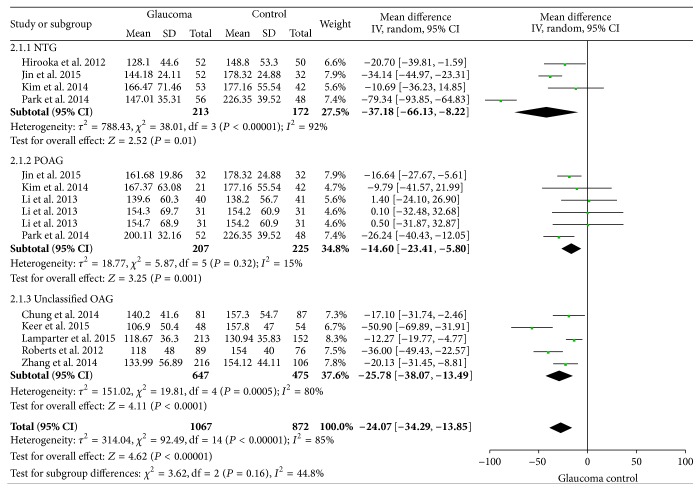
Forest plot of subgroup analysis of average PPCT.

Subgroup analysis was carried out according to the type of glaucoma and the result showed that there was a significant difference of average PPCT between POAG patients and controls (WMD = −14.60, 95% CI: −23.41, −5.80) with no heterogeneity (*I* ^2^ = 15%); similar result was observed in NTG patients (WMD = −37.18, 95% CI: −66.13, −8.22) but with significant heterogeneity (*I* ^2^ = 92%) (Figure 4). The data showed changes in PPCT appeared to be correlated with POAG as well as NTG. There was no sufficient data to conduct further analysis for PPCT in the 4 sectors.

### 3.4. Sensitivity Analysis

**Figure 5:**
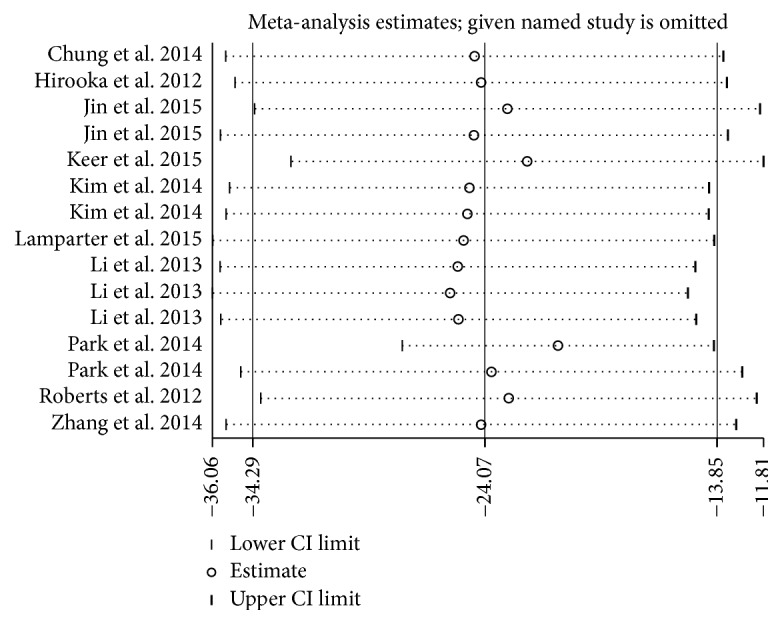
Sensitivity analysis of average PPCT. CI indicates confidence interval.

**Figure 6:**
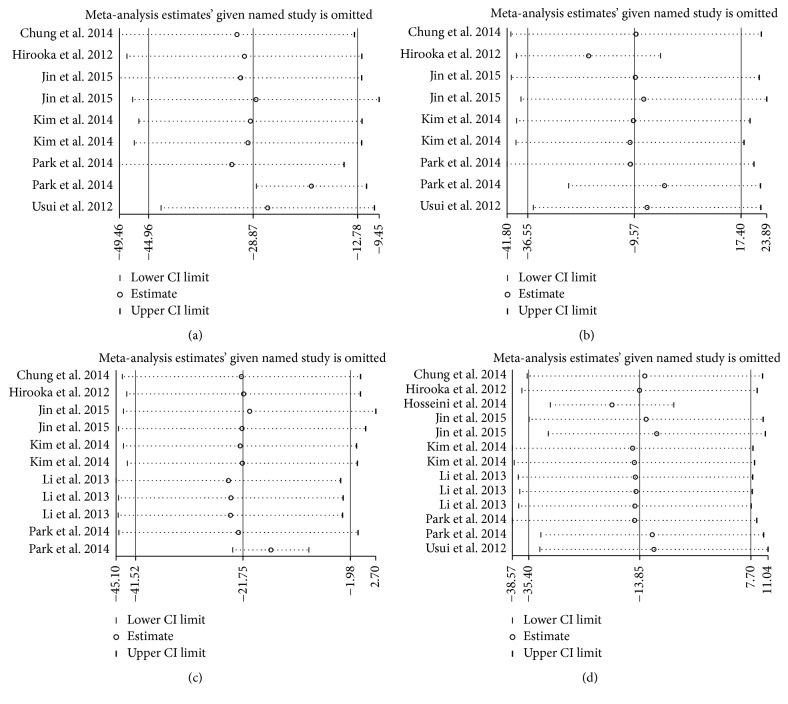
Sensitivity analysis of PPCT in each sector: (a) superior, (b) inferior, (c) nasal, and (d) temporal. CI indicates confidence interval.

Figures 5 and 6 were generated to evaluate the influence of a single study on the pooled results, and the results did not change significantly when any particular study was removed, which confirmed the stability of the results. Because of the small sample sizes, we did not conduct further sensitivity analyses in the subgroup analysis.

### 3.5. Publication Bias

**Figure 7:**
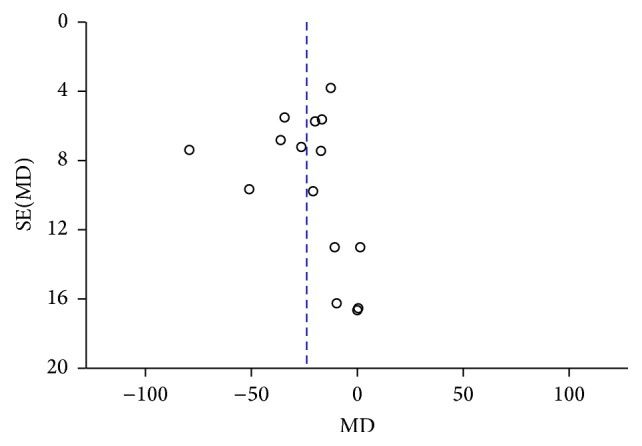
Funnel plots for evaluating the publication bias of average PPCT between the two groups.

**Figure 8:**
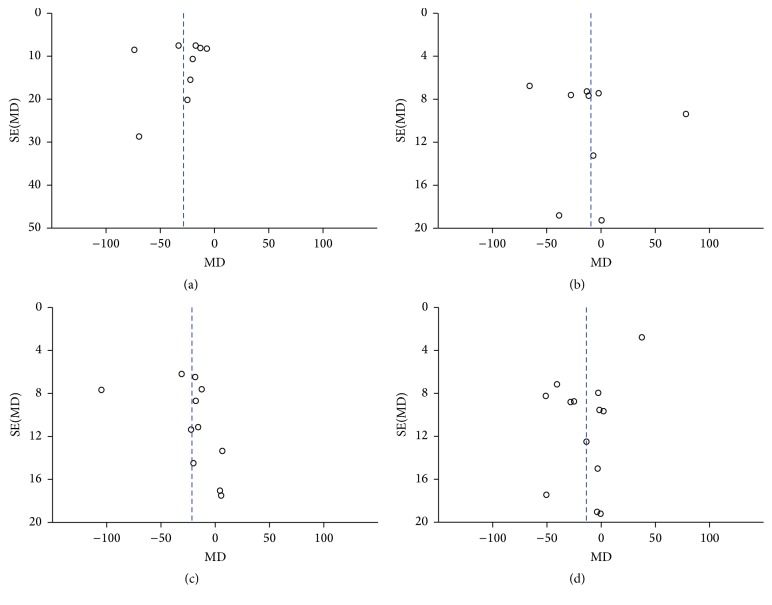
Funnel plots for evaluating the publication bias of PPCT in each sector: (a) superior, (b) inferior, (c) nasal, and (d) temporal.

To assess the publication bias of the literature for average peripapillary choroidal thickness, a funnel plot was displayed intuitively (Figure 7). Publication bias was also calculated using Begg's test (*P* = 0.499) and Egger's test (*P* = 0.859), and no obvious evidence of publication bias was found. Similar results were revealed in the analysis of each sector (Figure 8), which did not reveal any asymmetry. We did not conduct publication bias analyses in the subgroups analysis due to the small sample sizes.

## 4. Discussion

With the mounting clinical evidence indicating the involvement of the peripapillary choroid in glaucoma, it has become increasingly important to detect changes of the choroid. Optical coherence tomography is a useful method for investigating anatomical parameters of the choroid with high reliability and reproducibility [31, 32]. Although OCT could not provide the exact hemodynamic physiology of choroidal circulation flow, it gives us better visualization of the choroid compared to previous instruments by the application of an enhanced depth imaging (EDI) model [33, 34]. Thinner peripapillary choroidal thickness is thought to be the result of loss of innermost choroidal vasculature and may be an anatomic risk factor for open-angle glaucoma, contributing to the progression of optic neuropathy. The ability to quantify these peripapillary choroid changes may allow enhancement of current models of initiation and progression of glaucoma.

Despite a large amount of studies exploring the relationship between OAG and PPCT, it remains controversial. The data in this meta-analysis showed that the average PPCT in OAG was significantly reduced compared to healthy individuals which was a potential support of the vascular theory of glaucoma and suggested the retrobulbar ischemia might have an impact on the optic nerve head. Contrary to this, previous meta-analyses conducted by Wang and Zhang [16] and Zhang et al. [17] both demonstrated no correlation between PPCT and OAG.

Besides, we found that the choroid was thinner in the superior and nasal sectors of the optic disc in glaucoma eyes. However, several studies have reported thinnest PPCT in the inferior region in normal eyes and hypothesized that thinner choroid makes this area more vulnerable to glaucomatous ischemic damage, giving a possible explanation why glaucoma typically affects the inferior optic nerve area first [35–37]. As we all know, glaucoma is often manifested with focal optic disc damage; none of these included studies addressed the morphological patterns of optic disc damage which might be highly related with the choroidal thickness around the optic nerve head. Therefore, current knowledge does not seem to give an exact explanation. Further investigations focused on the relationship between the type of glaucomatous disc damage and the distribution of peripapillary choroidal thickness are required to address this problem.

Subgroup analysis revealed that glaucoma type had a close connection with PPCT which needed to be considered. Both POAG and NTG showed significant difference in average PPCT but with opposite heterogeneities, which probably indicated that reduction of choroidal thickness around the optic disc might play a part in the pathogenesis of NTG and POAG, just in accordance with previous published studies showing reduced peripapillary choroidal circulation in patients with POAG as well as NTG [38–40]. With more and more quantitative techniques becoming available, the debate over choroidal deficits in the pathophysiology of glaucoma will come to a consensus.

### 4.1. Strengths and Limitations of the Meta-Analysis

In contrast with the earlier meta-analyses conducted by Wang and Zhang [16] and Zhang et al. [17], we examined a wider range of clinically relevant outcome measures and focused on direct comparisons between OAG and healthy controls after extending the date of literature search by one year. Wang and Zhang included relatively limited studies (*n* = 6) and Zhang et al. actually included 10 studies when analyzing the relationship between OAG and PPCT. Three studies in the synthesis conducted by Zhang et al. [17] were not included in our analysis and the reasons were stated as follows. Hosseini et al. [22] only measured the PPCT at the point about 1000 microns from the temporal side of the optic disc border, roughly at the same location where the 3.46 mm circumpapillary RNFL measurement circle crosses the horizon linear scan. Such a specified location could not represent the average choroidal thickness around the optic disc, which may introduce bias in the synthesis. Suh et al. [14] examined 61 unilateral NTG patients and compared the PPCT of the glaucomatous eyes with the contralateral normal eyes. Maul et al. [12] reviewed 23 OAG patients and 30 OAG suspects. Since the latter two used those with high possibility to develop glaucoma as the control groups, it is possible that no association was identified. The above three were excluded and four refreshed studies were included after a stricter application of the inclusion criteria. What is more, another study conducted by Sigler et al. [15] was not included which just provided the mean value without the SD. They compared 24 eyes with POAG with 32 control eyes and found statistical thinning of average PPCT as well as the choroidal thickness in each quadrant in POAG patients. Finally, 13 studies involving 1067 eyes in the experiment group and 876 eyes in the healthy control group were included in our meta-analysis. Smaller sample sizes could increase the risk of making a falsely negative conclusion and apparently conclusive meta-analysis may be inconclusive. Increased sample sizes could have influenced the direction of the conclusion. Therefore, we do have reasons to believe the changes of PPCT might exist in OAG.

Although there are important discoveries revealed by these studies, there were several limitations in this meta-analysis. First, studies included in our meta-analysis examined patients with variable types of OCT instruments and different OCT provided different scan methods. Also, the segmentations of the choroid were performed manually and the measurements were conducted at different locations. The measurements may not display the whole dimensions of the choroid. Although we had made a significant effort to select the most consistent data for analysis, we still could not completely exclude the measurement bias. Second, there was substantial heterogeneity among studies in the primary analysis. Different OCT instruments, different measurement points, and patient characteristics, such as race, sex, age, and axial length, may contribute to the heterogeneities in our meta-analysis. We only conducted a subgroup analysis according to the type of glaucoma, which indicated that the type of glaucoma might be a risk factor influencing peripapillary choroidal thickness. The remaining factors were too various to perform subgroup analysis or meta-regression which might explain the heterogeneities to a certain extent. Third, only published studies were included and no apparent evidence of publication bias was presented in our analysis; however, unpublished studies and original data may be neglected; thus a potential publication bias may exist. Additionally, not all potentially confounding factors such as diurnal fluctuation of PPCT, medication treatments, and systematic vascular related diseases were reported among the included studies, which would increase the risk of bias. The results based on the quantitative research synthesis suggest that further researches are required to better describe the relationship between different glaucoma patients and peripapillary choroidal thickness in detail.

In a word, recent innovations in optical coherence tomography have helped to better visualize and quantitatively analyze the choroid effectively. But the association between the choroidal thickness measured by optical coherence tomography and the choroid circulation has not been completely understood. As the technology continues to evolve, the combination of OCT and angiography shows the potential to assess ocular hemodynamics and reveals that glaucomatous eyes have reduced peripapillary flow [41]. Though so many uncertain and unpredictable factors may be involved, OCT shows excellent prospects for the future research. Further ongoing advancements in technologies are desiderated and expected to explore the relationship between the choroid circulation and open-angle glaucoma in detail.

## 5. Conclusions

Our meta-analysis indicated that average peripapillary choroidal thickness decreased in open-angle glaucoma. The results also highlight that peripapillary choroidal thickness measured by optical coherence tomography may be an important parameter to consider in open-angle glaucoma.

## Supplementary Material

This meta-analysis was performed in accordance with the generally accepted methodology recommendations below and the PRISMA (Preferred Reporting Items for systematic Review and Meta-Analyses) statement was followed in the whole process.

## Figures and Tables

**Table 1:** Characteristics of the included studies.

Author (year)	Location	Glaucoma	OCT	Number of eyes		Mean age (year)		Mean axial length (mm)		IOP at imaging (mmHg)		Mean visual field MD (dB)	
				Case	Control	Case	Control	Case	Control	Case	Control	Case	Control
Hirooka et al. 2012 [24]	Japan	NTG	Heidelberg	52	50	66.2 ± 13.1	62.4 ± 10.0	24.9 ± 1.5	24.4 ± 1.4	12.8 ± 1.9	14.1 ± 2.8	−12.1 ± 7.3	NA
Roberts et al. 2012 [28]	Canada	OAG	Heidelberg	89	76	71.1	56.1	23.9	23.8	14.8	17.2	−3.4	+0.57
Usui et al. 2012 [25]	Japan	NTG	NA	12	12	33.6 ± 6.4	31.2 ± 4.1	27.6 ± 0.5	27.2 ± 0.5	13.7 ± 2.6	14.2 ± 2.4	−4.2 ± 3.7	−1.2 ± 0.9
Li et al. 2013 [26]	China	POAG	Heidelberg	31	31	57.2 ± 14.7	57.9 ± 14.6	NA	NA	16.4 ± 3.0	15.8 ± 2.3	9.20 ± 7.65	NA
Li et al. 2013 [26]	China	POAG	Heidelberg	31	31	57.2 ± 14.7	57.9 ± 14.6	NA	NA	16.6 ± 2.4	15.8 ± 2.3	−0.37 ± 0.99	NA
Li et al. 2013 [27]	China	POAG	Heidelberg	40	41	62.8 ± 10.7	62.1 ± 10.2	NA	NA	NA	NA	NA	NA
Hosseini et al. 2014 [22]	America	OAG	Cirrus Zeiss	58	33	67.8 ± 8.7	56.6 ± 9.5	24.9 ± 1.4	23.7 ± 0.8	13.4 ± 3.5	14.3 ± 2.6	−3.5 ± 3.5	−0.3 ± 2.0
Zhang et al. 2014 [23]	America	OAG	Topcon	216	106	71.82 ± 10.19	61.21 ± 11.89	24.13 ± 1.25	23.87 ± 1.00	14.02 ± 4.33	13.94 ± 2.79	−5.25 ± 6.29	0.13 ± 1.30
Park et al. 2014 [18]	Korea	NTG	Heidelberg	56	48	63.54 ± 13.63	57.78 ± 13.29	24.24 ± 2.74	23.56 ± 2.54	15.33 ± 1.86	13.78 ± 2.15	−5.06 ± 3.52	−0.23 ± 0.77
Park et al. 2014 [18]	Korea	POAG	Heidelberg	52	48	60.19 ± 14.10	57.78 ± 13.29	23.16 ± 2.38	23.56 ± 2.54	17.51 ± 3.26	13.78 ± 2.15	−5.26 ± 3.41	−0.23 ± 0.77
Chung et al. 2014 [19]	Korea	OAG	Heidelberg	81	87	53.9 ± 14.2	51.1 ± 11.1	24.7 ± 1.71	23.9 ± 0.97	15.4 ± 3.01	15.0 ± 2.86	−5.52 ± 5.78	−0.48 ± 1.42
Kim et al. 2014 [20]	Korea	NTG	Heidelberg	53	42	52.75 ± 16.60	49.13 ± 17.09	25.11 ± 1.43	24.70 ± 1.22	NA	NA	−2.21 ± 1.95	1.3 ± 1.10
Kim et al. 2014 [20]	Korea	POAG	Heidelberg	21	42	53.98 ± 11.23	49.13 ± 17.09	24.93 ± 1.52	24.70 ± 1.22	NA	NA	2.99 ± 1.63	1.3 ± 1.10
Lamparter et al. 2015 [29]	Germany	OAG	Optovue	213	152	65.39 ± 9	55.68 ± 13	NA	NA	16.52 ± 6.11	15.51 ± 2.78	NA	NA
Van Keer et al. 2015 [30]	Belgium	OAG	Cirrus Zeiss	48	54	72.0 ± 9.1	21.6 ± 1.1	NA	NA	12.2 ± 3.9	15.9 ± 3.0	−9.0 ± 8.1	NA
Jin et al. 2015 [21]	Korea	NTG	Optovue	52	32	58.64 ± 2.64	60.28 ± 4.28	24.01 ± 1.14	23.99 ± 1.04	13.84 ± 2.03	11.67 ± 1.49	−4.11 ± 1.85	−0.28 ± 0.11
Jin et al. 2015 [21]	Korea	POAG	Optovue	32	32	59.32 ± 3.35	60.28 ± 4.28	23.78 ± 1.02	23.99 ± 1.04	14.55 ± 2.54	11.67 ± 1.49	−4.22 ± 2.47	−0.28 ± 0.11

OAG indicates open-angle glaucoma, NTG indicates normal tension glaucoma, POAG indicates primary open-angle glaucoma, IOP indicates intraocular pressure, and NA indicates not available.

**Table 2:** Quality assessment of included studies using the Newcastle-Ottawa scale.

	Selection				Comparability	Exposure			Total score
	Case definition	Representativeness of the case	Selection of controls	Definition of controls	Comparability of cases and controls	Ascertainment of exposure	Same method of ascertainment for cases and controls	Nonresponse rate	
Hirooka et al. 2012 [24]	☆	☆		☆	☆☆	☆	☆		7
Roberts et al. 2012 [28]	☆			☆	☆☆	☆	☆		6
Usui et al. 2012 [25]	☆	☆		☆	☆☆	☆	☆		7
Li et al. 2013 [26]	☆			☆	☆☆	☆	☆		6
Li et al. 2013 [27]	☆			☆	☆☆	☆	☆		6
Hosseini et al. 2014 [22]	☆			☆	☆☆	☆	☆		6
Zhang et al. 2014 [23]	☆			☆	☆☆	☆	☆		6
Park et al. 2014 [18]	☆	☆		☆	☆☆	☆	☆		7
Chung et al. 2014 [19]	☆	☆		☆	☆☆	☆	☆		7
Kim et al. 2014 [20]	☆	☆		☆	☆☆	☆	☆		7
Lamparter et al. 2015 [29]	☆	☆		☆	☆☆	☆	☆		7
Van Keer et al. 2015 [30]	☆			☆	☆☆	☆	☆		6
Jin et al. 2015 [21]	☆	☆		☆	☆☆	☆	☆		7

One ☆ represents one score and the maximum score is 9.
